# Achieving best outcomes of patients with cardiovascular diseases in China by enhancing the quality of medical care and establishing a learning health care system

**DOI:** 10.1016/S0140-6736(15)00343-8

**Published:** 2015-10-10

**Authors:** Lixin Jiang, Harlan M Krumholz, Xi Li, Jing Li, Shengshou Hu

**Affiliations:** National Clinical Research Center of Cardiovascular Diseases, State Key Laboratory of Cardiovascular Disease (LJ, XL, JL, SH), Fuwai Hospital, National Center for Cardiovascular Diseases, Chinese Academy of Medical Sciences and Peking Union Medical College, Beijing, People's Republic of China; Section of Cardiovascular Medicine and the Robert Wood Johnson Foundation Clinical Scholars Program, Department of Internal Medicine, Yale University School of Medicine; Department of Health Policy and Management, Yale School of Public Health; Center for Outcomes Research and Evaluation, Yale-New Haven Hospital (HMK), New Haven, Connecticut, United States

## Abstract

China faces the immediate need of addressing the rapidly growing population with cardiovascular disease (CVD) events and the increasing numbers who are living with CVD. Despite progress in increasing access to services, China faces the dual challenge of addressing gaps in quality of care and producing more evidence to support clinical practice. In this article, we address opportunities to strengthen performance measurement, programs to improve quality of care and national capacity to produce high impact knowledge for clinical practice. Moreover, we propose recommendations, with implications for other conditions, for how China can immediately leverage its *Hospital Quality Monitoring System* and other existing national platforms to evaluate and improve performance, as well as generate new knowledge to inform clinical decisions and national policies.

## Introduction

China is experiencing a rapid epidemiological transition, with particular implications for the growth of cardiovascular disease (CVD).^1^ From 1990 to 2010, CVD as a cause of death increased from about 25% to 40%.^2^ Moreover, even now the population prevalence of CVD is high, with estimates of 290 million individuals being affected by CVD. With the effects of changing lifestyles and an aging population, the growth in the numbers of individuals with CVD is predicted to continue at least till 2030. In this time period, the numbers of people with acute myocardial infarction (AMI) is estimated to increase from 8.1 to 22.6 million a year, and those with stroke from 8.2 to 31.8 million a year.^3,4^ Although preventive strategies are the ultimate solution to this epidemic of CVD, including attention to environment and behaviours,^5^ China faces the immediate need of caring for the rapidly growing population who are having and will have CVD events and the increasing number of individuals who are living with CVD. Moreover, these challenges are also relevant to many other non-communicable disease conditions.

China has recently been strengthening its health care system through far-reaching health care reform policies, focusing on insurance coverage,^6^ hospital capacity,^7^ and the health care workforce,^8^ and has made much progress in expanding access to affordable care.^9^ However, even with these advances, China has additional work to do to ensure that these individuals have access to care that best enables them to achieve best possible health outcomes.

As errors in medical care have caused numerous deaths and disabilities in low- and middle-income countries, which lack evidence about which strategies work best in resource poor settings.^10^ In particular, there are two areas that are vital to China's ability to meet the health needs of individuals who suffer from CVD (Panel 1). First, there is a need to improve the quality of care. Building health services capacity and fostering access are necessary, but not sufficient to ensure that individuals benefit from health care services. They must have access to the highest quality care. Second, there is a need to expand the evidence about the safety and efficacy of treatments for Chinese patients and how best to deliver the highest quality care. To achieve these goals, China needs to build a learning health care system, with the capacity to monitor performance, learn about what works best for whom, and evaluate what strategies support successful implementation of best practices and achieve optimal outcomes. In this way, China can be a model in showing how to make its health care system more accessible, but also configure it to deliver high quality care and to learn from the experience of every patient.

## Quality of CVD Care

In a health system with high quality of care, patients get the care they need, when they need it, without undergoing unnecessary or inappropriate treatments.^11^ High quality care not only provides patients with the best opportunity to achieve the outcomes they seek, but avoids inefficiency and waste. Countries with limited resources particularly need to focus on what care is best and how it is provided,^10^ and strengthen healthcare delivery systems so they can produce high-level performance as efficiently as possible.

### Gaps in Performance in CVD Care

Prior studies and government reports from China indicate large gaps in quality. We did a comprehensive literature search, focusing on quality of cardiovascular care, including the themes of healthcare quality noted by the US Institute of Medicine and World Health Organization (Appendix 1, Appendix 2).^11,12^ We found evidence of progress in the care of people with coronary heart disease (CHD) and stroke, but also substantial opportunities for improving quality of CVD care (Appendix 3).

One national representative study of patients with ST-segment elevation myocardial infarction (STEMI) found that in-hospital mortality rates, adjusted for demographic and clinical factors, have not improved from 2001 to 2011,^13^ a period when many other countries experienced marked declines.^14,15^ The lack of improvement in China may be a result of no change in the underuse of reperfusion therapy, beta-blockers, and angiotensin converting enzyme (ACE) inhibitors. In particular, the reperfusion therapy rates were much lower than many other countries. For example, in a recent period China achieved 27% treated,^13^ compared with 94% in the United States (US),^16^ 77% in the United Kingdom (UK),^15^ and 59% in India.^17^ Even among those patients who were ideal candidates for treatment, only half of them received the therapy. In addition, the effectiveness of treatment was compromised because of delays in treatment, with door-to-needle and door-to-balloon times for reperfusion much longer than the recommended standards.^18,19^ Similar findings have shown underuse and delay is common in administration of fibrinolytic therapy in patients with acute ischemic stroke (AIS).^20^

The issues with quality are not restricted only to acute treatment, as there are also gaps in secondary prevention, even as clopidogrel and statin treatment rates increased. According to the baseline characteristics of randomized participants of an international trial, HPS2-THRIVE, the use of statins among 10 932 Chinese participants was much lower than that among 14 741 European patients (48% vs 96%), who were similarly at high risk of vascular disease and had definite indicators for statins.^21^ Moreover, among Chinese hypertensive patients with history of ischaemic stroke, only 21% had their blood pressure controlled, as defined as lower than 140/90 mmHg for non-diabetic patients and 130/85 mmHg for diabetic patients.^22^ This underuse of secondary preventive medications is consistent across several studies. ^23-30^

Moreover, the studies show that not only do many people with strong indications for treatment not get treated, but there is also frequent treatment of people with contraindications. In AMI care, the use of beta blockers in patients at high risk for cardiogenic shock, which is considered a strong contraindication, was common, with 43.7%, 59.6%, and 53.6% of these patients receiving treatment in 2001, 2006, and 2011 respectively. Thus, there was no decline in the unsafe practice pattern of beta blocker use even after it was highlighted in the landmark trial in Chinese patients more than a decade ago,^31^ and a warning incorporated into international guidelines.^32,33^

Misuse of treatments has also been documented, undermining their benefit. For example, among patients with STEMI who were treated with fibrinolytic therapy, in addition to significant delays in administration. Also, based on guideline recommendations, it was commonly underdosed.^34,35^

The average quality performance across China obscures the fact that quality varies by hospitals and regions. In a study of a large network of hospitals that perform coronary artery bypass grafting (CABG) the in-hospital risk-standardized mortality rates in the years 2007 and 2008 ranged more than five-fold, from 0.7% to 5.8%. Regional differences in CABG mortality ranged from 1.6% in Eastern region to 2.5% in Central region.^36^ Hospitals also varied in their use of guideline-recommended medications. Although 90% of eligible patients with AMI received early aspirin treatment in 2011 overall, many hospitals lagged in performance, with 15% treating fewer than 80% of their patients with aspirin.^37^

The reason that quality of care is not higher is not known. The substantial underuse of beneficial treatments cannot be explained easily as some strategies were adopted quickly and others were not. Moreover, the quality gaps not only exist in the expensive treatments that need advanced techniques and facilities (e.g., CABG), but also in the use of inexpensive treatments, which are accessible and simply administered (e.g., beta blockers and aspirin).

Adding to the complexity is the dual role of hospitals in China, as they typically act as the mainstay of both inpatient and outpatient care. Furthermore, data on the performance of other potential care settings such as primary care centres, is largely unavailable and hinders assessment and improvement in these areas, highlighting the need to identify strategies to collect and learn from the care provided in these settings.

### Inadequate measurement and incentives

Continuous quality improvement is a cycle of identifying targets, intervening to improve, and assessing the effects. Only by measuring and reporting clinical practice measures, which are constructed with appropriate and standard criteria, can hospitals and health professionals clearly understand their opportunities for improvement. In the end, measurement is critically important, but it is also important to recognize that the objective is not measurement, but improvement leading to exemplary performance. Therefore, the measures need to be timely and actionable, so that health care organizations and individuals can respond quickly. Although China has made some achievements in developing a national system to collect clinical information, quality measurements have yet to be used as a catalyst to improvement.

The *Hospital Quality Monitoring System* (HQMS), an online data collection system launched in 2011, covers all tertiary hospitals over the country and can provide a platform for measurement and improvement. The system is designed to link hospital information systems and to upload a common dataset of information from all inpatients medical records (Table 1) (Appendix 4). The HQMS, as the first system that can electronically capture data from each tertiary hospital, is supposed to capture data for all inpatients automatically. However, HQMS lacks clinical information about treatments and patient indications and contraindications, which can contribute to performance indicators such as the use and timeliness of reperfusion therapy in patients with AMI. Nevertheless, despite these limitations, the basic information is comparable to the claims data required by the Centres for Medicare & Medicaid Services in the US, which is used for national hospital performance evaluation and public reporting – and as a component of national incentive programs.^14^ In China, HQMS is not yet used to produce national quality metrics, leaving its potential role as a stimulus for quality improvement unfulfilled.

The Chinese government issued standard clinical pathways for each of 120 common admission diagnoses, including 11 CVD categories, and established an official monitoring system in 2009 for these pathways. This system, the *Clinical Pathway Report for Specific Diseases* (CPRSD),^38^ seeks to standardize the treatment processes, enhance the management of cost and length of stay (LOS) and support the development of prospective payment systems in the future. For each inpatient case doctors are required to complete a detailed clinical pathway checklist (Appendix 5). The hospitals, however, are only required to provide the government with a brief summary of these reports every three months, rather than provide the patient-level data in a form that is amenable to detailed quality reporting. Therefore, these data have not yet been used for national quality reporting. Moreover, there is currently no auditing to assess the accuracy of the data.

As a consequence of not producing national information on performance, there is no mechanism to benchmark performance in a systematic way and to link incentives for performance with the interests of care providers in China (Table 1). For hospitals, after the HQMS was established in 2011, new official standards for tertiary or secondary hospitals were issued,^39,40^ with a majority of the core criteria related to patient safety and care quality, also with indicators of readmission and reoperation after specific diseases like AMI and AIS embedded. However, without risk adjustment, the usefulness of the comparisons are limited because of the possibility that differences are the result of variation in case mix.

Despite some regional experiments and pilot projects, the payments to providers in China are still based on the number of care services provided, and not whether those services are appropriate or performed well – or whether good outcomes are achieved.^41^ The mainly fee-for-service payment provides an incentive for the pursuit of treatment volume, rather than quality, and can lead to prescribing profitable treatments and tests that may not be clinically needed,^42,43^ as has been noted in other countries.^44^ Thus, to align incentives with the best interests of patients is needed in China. Such incentives should promote prevention, evidence-based care and value. There is a particular need to reduce the waste of resources in a system that must serve more than a billion people.

In summary, we identify a need to strengthen performance measurement, accountability mechanisms, and alignment of reward structures with the provision of high-quality, high-value care. Hospitals and doctors have little information about their performance and lack knowledge about what are the major gaps, how they can best improve, or how these interventions can be assessed and acknowledged, also the system lacks imperative motivation for delivering high quality service. Thus, there is an opportunity for the system to improve and respond to these needs. Moreover, transparent and accountable quality of care is crucial for the trust of patients in the performance of hospitals and doctors. This issue may be particularly important in China where the hospitals and doctors are experiencing an unprecedented crisis of confidence.^45,46^ Without measurement and quality accountability, patients may ask: how can I have faith in my doctor or hospital? With incentives that may be at cross-purposes to high-quality, high-value care for me, how can I trust the health care system to promote my interests? Attention to quality may be needed to address the growing tension between doctors and patients.^46^

### Efforts in Quality Measurement and Improvement

Despite the challenges, there are formative efforts to improve the quality of care. To strengthen quality management and standardize clinical care, China has been drafting the *Medical Quality Management Regulations*,^47^ a program in which some tertiary hospitals will be appointed as the national or regional *Medical Quality Control Centres* (MQCC) in specific clinical fields. The intent for these Centres is to take the lead in formulating quality standards, collecting data, performing analyses, and disseminating findings for the purpose of quality control and improvement. With this effort, the professional communities in China then could become more engaged in the care quality management in addition to strong government leadership, which is considered critical according to experiences from the US and the UK, despite their different healthcare systems.

Clinical research focusing on quality and implementation science can provide valuable insights for quality improvement. For example, studies based on a national representative hospital network, China Patient-centred Evaluative Assessment of Cardiac Events (PEACE), and a national CABG registry, Chinese Cardiac Surgery Registry (CCSR), have identified clear targets for quality improvement.^13,36^ More importantly, these studies have developed collaborative and reusable platforms, which are poised for translating available evidence into optimal clinical care for CVD and continue in continuous data collection and evaluation. Such studies can produce interventions that can be tested in trials, such as the current ongoing cluster randomized trial, PEACE-2. In another example, the Clinical Pathways for Acute Coronary Syndromes (CPACS) group developed and evaluated specific clinical pathways and corresponding quality improvement strategies for ACS, including regular feedback and audit on key performance indicators.^48^ The studies found that despite the improvement in use of discharge medicines, there was no significant difference in early treatments, which need more coordination and have stronger effects on reducing early mortality. The CPACS group identified barriers to implementation of the clinical pathway,^49^ including lack of leadership support, variation in the capacity, healthcare funding constraints, are instructive for the future relevant efforts. Several other studies are in process and will provide further insight on quality improvement strategies for CVD care.^50,51^

## Evidence for Practice

### Paucity of Evidence in CVD Care

In addition to the need to better apply existing knowledge, China urgently needs to expand the evidence for what works best for whom. First, there is an overall deficit of information even in an evidence-rich area such as cardiovascular disease. As much as half of guideline recommendations are based on expert opinion. Beyond that, much of the medical evidence used in China is imported, with a paucity of local evidence about effectiveness and safety of treatments, even as clinical research in the country has grown rapidly during the past decades. There is evidence that Chinese patients may react to medications differently in effectiveness and safety from other populations.^21,52^ For example, the risk of myopathy in Chinese patients taking daily 40 mg simvastatin is ten times higher than in Caucasians.^21^ Moreover, the system is different, as is the use of traditional Chinese medicine, which may interact with certain medications or affect particular strategies.

**Figure 1:**
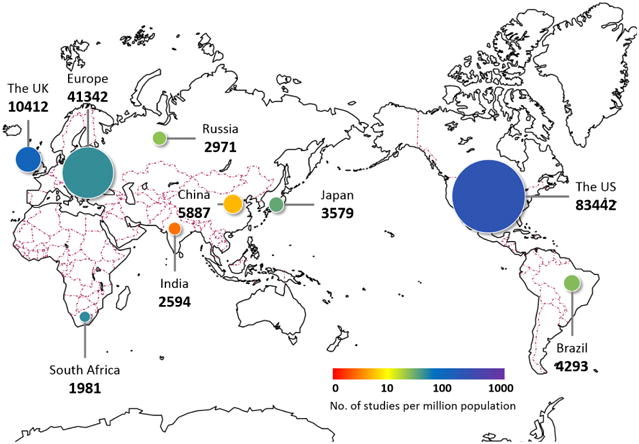
A global map of clinical studies registered in clinicaltrials.gov in major economies: Multinational studies were counted in each of involved countries

China under-produces medical knowledge compared with many other countries. With respect to the number of clinical studies registered in a worldwide registry database (www.clinicaltrials.gov) of the US National Institutes of Health, China (5.9 thousand) lags far behind high-income countries, such as the US (83.4 thousand) and the UK (10.4 thousand). Moreover, despite having the world's largest population with over 1.3 billion, the number of studies per million people in China is disproportionally smaller than South Africa, Japan, Brazil, and Russia, as well as the global average level (Figure 1). In the 575 randomized clinical trials on cardiovascular diseases conducted in China, less than one fifth (112 studies) recruited over 1000 patients, with the primary comparison based on health outcomes, and a minority of them (46 studies) were sponsored by Chinese organizations.

Consequently, China's clinical practice recommendations are largely derived from expert opinions, low-level evidence, or imported knowledge. Professional societies in China have released 14 Clinical Practice Guidelines (CPGs) for CVD management in China, while there are 186 Expert Consensuses (ECs). The ratio (14 CPGs vs 186 ECs) is much lower than the current ones from American Heart Association (55 CPGs vs 16 ECs)^53^, European Society of Cardiology (35 CPGs vs 13 ECs), or National Institute for Health and Care Excellence (13 CPGs vs 4 ECs). There are 216 recommendations with the evidence level A (highest) in Chinese CPGs,^54-62^ among which only less than 10% are based on studies that involved Chinese patients.

### Insufficient capacity for clinical research

An overall strategic plan for clinical research in China has yet to be established for several reasons. First, despite the increasing investments for medical research from the Chinese government, funding for clinical research was only one fourth of that for basic biomedical research ($250 million versus $1 billion).^63,64^ Second, there are human capital issues. Training of clinical research design and implementation is weak in China. Most clinical research largely relies on individual or small groups of practicing doctors with limited time for investigation, which affects the development of studies in China, let alone the requirements for cooperation across scientific fields of statistics, information techniques, sociology, economics and others. Third, without continuous funding support, clinical research networks for multicentre studies are often organized for single projects in China, leading to one time use and wasting the opportunity to reuse them. Moreover, they lack components that would provide the necessary training and mutual communication in implementation. Finally, the regulatory system needs to be more proactive in identifying identify opportunities to promote conducting clinical trials in China.^65^

### Efforts to strengthen clinical research

The Chinese government has initiated long-term investments in clinical research that will strengthen the country's ability to generate evidence for clinical practice and policy formulation. In 2013, the Chinese Ministry of Science and Technology and National Commission for Health and Family Planning officially announced the funding of Chinese National Clinical Research Centres (NCRC).^66^ At this point, 19 NCRC were accredited, covering 9 clinical disciplines, including all major non-communicable diseases. The government supports NCRC to establish sustainable collaborative networks, to take leading roles in multicentre clinical studies on knowledge generation, translation and application, and more importantly, to participate in the formulation of national clinical research roadmap and strategies. The vision for the NCRCs is to promote clinical research that can be translated into practice, to strengthen development, improvement and use of high value health care services, to reduce inefficiency and wasteful practices, and to improve population health and clinical outcomes. From the government, staged investments have underscored the missions of the NCRC – the first round funding of CNY81 million (about US$13 million) in 2014, has supported 13 clinical studies, one of which was to assess the quality improvement interventions focusing on gaps recently identified in China PEACE study and CCSR; the second round of CNY320 million (about US$52 million) in 2015, is about to support 27 studies on “assessments of management strategies for major diseases”. These efforts are expected to lay a foundation for future clinical evidence generation in China.

## Suggestions for Improvements in CVD Care

To improve the care and outcomes of patients with CVD, it is essential for China to further extend previous efforts to generate the knowledge of what are best practices in its own context, to measure whether doctors and hospitals are applying this knowledge in practice, to reward excellent performance, and to refine strategies and tools that will assist performance. The strategy needs to involve two major aspects: 1) evaluating through measurement, improving through stronger systems; 2) learning through research directed at what practices work best for which patients and integration with clinical care providers to promote the translation of the practical science into efforts to strengthen systems so that they have exemplary performance.

### Quality measures, disseminations and incentives for evidence application

China needs better translation of clinical, outcomes and implementation research to the clinical setting and measures of performance that can promote quality improvement, greater transparency in performance, and better policies to reward value, so that every citizen has opportunity to be treated according to the highest-quality care.

China needs efficient systems for continuously and timely monitoring nationwide clinical practice. Ideally these systems could build on existing efforts. There are two existing platforms that could be leveraged. First, the HQMS, with a potential expansion to secondary and even primary hospitals in future, could be fostered as a national administrative claims database. In addition, its nationwide unique patient identification system allows linkage across time and venues. It can be used for not only descriptions of hospital utilization, but also quality assessments through risk-standardized outcomes measures, as the US Centres for Medicare & Medicaid Service does, serving to inform the public of hospital performance and to support policy initiatives to improve care.^67^ Over time a national health information exchange can provide for higher quality clinical data to be shared securely to promote better measures and research in the service of better care.

To optimize the use of the HQMS, it will be useful to strengthen the system in several ways: 1) systems to assess, standardize and improve data quality will be important; 2) consideration of the addition of limited clinical data, as is in process in the US, would strengthen the utility of the database for risk adjustment, with accurate unique identifier allowing for record linkage; 3) the linkage of HQMS with other national databases such as death registry and insurance, can allow the assessment of mortality in standardized periods of follow up. Assessing out-of-hospital mortality is particularly important in China because many people leave the hospital just before death. In a universal public insurance system covering 96% of the population established in China,^68,69^ there is information about what test and treatments were paid for that is available to the central and local governments.^6,9^ This information can also be linked with the HQMS via insurance ID and social ID of patients to enable more detailed assessments of the patterns of care.

Second, the existing CPRSD in China, as well as the platforms of China PEACE and CCSR, as basis of national clinical registries focusing on quality improvement, can provide detailed clinical information for treatment patterns and outcomes assessment for specific diseases. The goal is to aggregate these data from hospitals across China and use them as part of the national quality assessment and improvement efforts. Currently the patient-level data in CPRSD are retained within each hospital – and yet these data, once combined, would provide detailed information on national practice. Concomitantly there is a need to invest in data quality. Also, the national and regional MQCCs can establish and continually refine the standardized data definition, operational instruction, and quality indicators through close collaboration with professional communities. These efforts should leverage existing registries, which have successfully developed standards, such as the US National Cardiovascular Data Registry (NCDR). Such alignment will enable the international comparison on quality of care for major CVD, which is of value for benchmarking performance and developing improvement interventions.

Furthermore, to achieve more efficiencies in data collection, it is possible to merge different data capturing systems in order to reduce redundancy. Moreover, China can develop innovative ways via information technology to directly capture clinical information of every healthcare encounter from electronic medical records in real time. The goal of common data standards and interoperability should be a national priority so the quality of data that can be shared can improve over time.

The performance of each hospital on key quality indicators should be publicly available, with national or regional comparisons and benchmarks. Transparency in measurement can provide a strong impetus for improvement. In the US, the introduction of a series of quality measures describing evidence-based care processes in AMI management in the 1990s revealed substantial variation.^14^ Policymakers responded with the strategies of mandating the recording of the measures and making them publicly available, which enabled performance on process measures, for instance door-to-balloon times, to quickly improve.^70^ Later, outcomes measures were introduced. This improvement in the quality of cardiovascular care in the US is undoubtedly an important driver of the profound decline in AMI mortality in recent years (Table 2).^102^

The government can seek to influence practice and promote implementation of best practices by means of incentives, economic and otherwise. Nevertheless, the effectiveness of such interventions in China are not known and it is necessary to ensure the designs do not cause incentives for adverse risk selection and other unintended consequences, as have been noted before.^71,72^ Therefore, evaluations of interventions need to be designed and implement from the outset.

### A learning health care system for evidence generation

Better evidence is not enough as China lacks a strong enough focus on implementation science to guide health care system design and to develop and evaluate policies and practices to ensure the full translation of evidence into clinical care. Implementation research remains in a very formative phase, and so little is known about how best to deliver care, especially across diverse venues of a massive country like China, where marked geographic variation in capacity and expertise exist. Implementation science lays the groundwork to learn from practice, including the post-approval experience with drugs and devices in Chinese populations, and then apply that knowledge to improvements in health care delivery. This research is essential to address the continuing need to determine what strategies work in which populations and how best to ensure that care is optimally delivered.

There are several feasible steps to achieve this vision. The databases can be made research ready by instituting systems to evaluate and promote the quality of the data. There also need to be incentives and accountability for data quality. Independent, random audits and the development of online tools to identify and correct errors can be instituted. One crucial step along the way is to build structured electronic medical record systems with unified standards for variable definition and coding, enabling research data collection, integration, and interoperability.^73^ In the future mHealth applications can play a more important role. To capitalize on this possibility there is a need to link the data by the unique identifier, which should be associated with every database – and every device, in which the mobile technology could extend the scope of data collection and feedback on care and outcomes. The linkage of the data, with the ability to track utilization and outcomes over time is critical to research and will enable a wide range of needed investigations and the possibility of new approaches to clinical monitoring and care.

For the applied clinical research infrastructure, the country has already taken a first step with the funding of the Chinese NCRC. The expectation is that there will be more multidiscipline teams and sustainable networks in the future. The core strength of this approach is that it leads to the development and evaluation of new strategies, which are the second half of quality improvement feedback loop. By engaging people, leveraging data embedded in daily clinical practice, and applying information technologies, a learning health care system will be fostered.^74^

## Conclusion

China has made remarkable recent advances in modernizing its health care system.^75,76^ Since 2003, China has achieved substantial improvement in access to and use of health services, as well as in hospital capacity. However, the growing access to services needs to be accompanied by marked improvements in quality. Achieving the best possible outcomes will require pragmatic efforts to improve the quality of care and generate useful evidence through the achievement of a learning health care system. The experience with CVD can serve as a model for other conditions. Moreover, the work in China can serve as a model of moving to a learning health care system where every individual receives care based on best practices and every experience is used to strengthen the system and advance knowledge for the future. China can achieve this vision through a coordination of efforts, a commitment to transparency around performance, an investment in practical research, a leveraging of advances in technology and a focus on the experience of each individual who seeks care from health care professionals and the health care system.

## Figures and Tables

**Table 1:** Quality measures for AMI and AIS across different countries

	China	The US	The UK
**Quality indicators**			
For AMI management	**Quality Control Indicators for Six Specific Diseases**^77^ Aspirin at presentation (clopidogrel if any contraindications) Left ventricular function assessment Reperfusion therapy (for STEMI only) (Door-to-needle less than 30 min; Door-to-balloon less than 90 min; Transfer out for primary PCI treatment) β blockers at admission Aspirin, β blockers, ACE inhibitors/ARB, and statins during hospitalization Aspirin, β blockers, ACE inhibitors/ARB, and statins at discharge Health educations Hospital LOS and total cost **Indicators of Medical Quality Management and Control for Tertiary General Hospitals**^40^ In-hospital mortality Readmission within 31 days	**Cooperative Cardiovascular Project**^78^ Reperfusion within 12h of arrival Aspirin during hospitalization Aspirin at discharge Beta-blocker at discharge ACE inhibitors at discharge Avoidance of calcium channel blockers at discharge Smoking cessation advice **Hospital Compare**^79^ Median time to transfer to another facility for acute coronary intervention Median time to ECG Fibrinolytic therapy within 30 minutes of arrival Aspirin at admission PCI received within 120 minutes of hospital Aspirin at discharge Statin at discharge ACE inhibitor for left ventricular dysfunction Beta-blocker at admission Beta-blocker at discharge Smoking cessation advice	**NICE Quality Measures**^80^ Diagnosis using the criteria in the universal definition Assessment of the risk using an established risk scoring system in patients with non-STEMI or unstable angina Length of time taken for intermediate or higher risk patients with non-STEMI or unstable angina to receive coronary angiography after admission. Length of time taken for adults with non-STEMI or unstable angina who are clinically unstable to receive coronary angiography. Coronary angiography for patients who were unconscious after cardiac arrest caused by suspected acute STEMI. Patients with acute STEMI who present within 12 hours of onset of symptoms receive primary PCI within 120 minutes of when fibrinolysis could have been given. **NSF for CHD performance indicators**^81^ Patients eligible for thrombolysis receiving it within 60 minutes of call for professional help. Beta blocker at discharge. Death during index admission for patients aged 35 to 74 years. Death within 30 days of infarct for patients aged 35 to 74 years.
For acute ischemic stroke management	**Quality Control Indicators for Six Specific Diseases**^77^ Reception procedure (follow the procedure; NIHSS assessment; CT scan, complete blood count),biochemistry, and coagulation function tests within 45 min Anticoagulant treatment for patients with atrial fibrillation Evaluation for t-PA or urokinase Aspirin or clopidogrel within 48 hours after admission Lipid profile assessment Dysphagia assessment Prevention for DVT Aspirin or clopidogrel at discharge Health educations Vascular function assessment within 24 hours after admission Hospital LOS and total cost **Indicators of Medical Quality Management and Control for Tertiary General Hospitals**^40^ In-hospital mortality Readmission within 31 days	**GWTG-Stroke Quality of Care Measures**^82^ IV rt-PA within 3 h of symptom onset Complications, IV rt-PA Early antithrombotic DVT prophylaxis Antithrombotic therapy at discharge Anticoagulation agents at discharge for AF Smoking cessation advice LDL cholesterol measured Lipid-lowering agent at discharge if LDL level is >100 mg/dl documented or patient taking lipid-lowering agents on admission Lipid-lowering agent prescribed at discharge for all patients except those with untreated levels of LDL <100 mg/dl. Weight management Diabetes management **Hospital Compare**^79^ Thrombolytic therapy with 3 hours after symptoms started Antithrombotic therapy by end of hospital day 2 DVT prophylaxis Discharged on antithrombotic therapy Anticoagulation therapy for atrial fibrillation/flutter Discharged on statin medication Stroke education Assessed for rehabilitation	**NICE Stroke Quality Standard**^83^ Screened for stroke or TIA outside hospital by ambulance staff Brain imaging within 1 hour of arrival at the hospital Admitted directly to a specialist acute stroke unit and assessed for thrombolysis Receive thrombolysis after assessment Swallowing screened within 4 hours of admission to hospital, before being given any oral food, fluid or medication Assessed and managed by stroke nursing staff and at least one member of the specialist rehabilitation team within 24 hours of admission to hospital. Assessed and managed by all relevant members of the specialist rehabilitation team within 72 hours of admission to hospital. Documented multidisciplinary goals agreed within 5 days of admission to hospital Ongoing inpatient rehabilitation after completion of their acute diagnosis and treatment who are treated in a specialist stroke rehabilitation unit. 45 minutes of each active therapy Reassessed and a treatment plan implemented for patients with loss of bladder control Screen for mood disturbance and cognitive impairment. Followed up within 72 hours for assessment and ongoing management for patients discharged with residual stroke-related problems Get care from those with clear information and management plan, as well as sufficient practical training
**Measure data systems**	**HQMS**: an official data collection system covering all tertiary hospitals, requires to upload a dataset of face sheet information in all inpatients medical records everyday, which includes patients' social ID, demographic characteristics, length of hospital stay, principal admission diagnosis, discharge diagnosis (principal ones and comorbidities), special treatments (procedures and surgeries), patient outcomes (death, non-recovery, improvement, or complete recovery), in-hospital infection (whether or not), transfusion, and the fee for the inpatient management	**Hospital Compare**: a national initiative covering over 4000 Medicare-certified hospitals, collects inpatient information for certain conditions including AMI and stroke, from over 40 million people age 65 and older. **NCDR ACTION-GWTG**: a national registry on AMI funded by American College of Cardiology/American Heart Association. It helps hospitals apply clinical guideline recommendations in their facilities and provides tools to measure care and achieve quality improvement goals. **GWTG-Stroke**: a national stroke registry and quality improvement program funded by the American Heart Association. Since its initiation in 2003, 1656 hospitals have entered more than two million patient records into the database. **Paul Coverdell National Acute Stroke Registry**: a national registry on acute stroke covering over 300 hospitals in 11 states, which is funded by the US Centers for Disease Control.	**MINAP**: a national registry on AMI began in late 1998, covering hospitals, ambulance services and cardiac networks in England and Wales. The data it collected allowed clinicians to examine the management of myocardial infarction within their hospitals against targets specified by the NSF for CHD. MINAP is the first national audit to release annual reports showing hospital performance against NSF targets in the public domain. **SINAP/SSNAP**: SINAP was a national clinical registry which collected information from hospitals in England about the first 72 hours of acute stroke care. Its results showing performance against important aspects of acute stroke care including 12 key stroke indicators were made public. SSNAP has superceded the SINAP since December 2012, and now is the single source of stroke data nationally, which collects a minimum dataset for every stroke patient.
**Quality accountability mechanisms**	None	Hospital Value-Based Purchasing is part of the Centres CMS' long-standing effort to link Medicare's payment system to a value-based system across the country.^84^ If a hospital's risk-adjusted readmission rate for certain patients exceeds the average level, CMS penalizes it in the following year for all Medicare admissions in proportion to its rate of excess re-hospitalizations of patients for the target conditions.^85^ CMS will continue to conduct regulation and enforcement activities to ensure that Medicare hospitals comply with federal standards for patient health and safety and quality of care.^86^	Hospitals are required to participate in national audit. NHS is planning to incentivize good quality care, like paying a higher tariff for more rapid angiography for non-STEMI

ACE indicates angiotensin converting enzyme, AMI acute myocardial infarction, ARB angiotensin receptor blocker, CHD coronary heart disease, CMS Centers for Medicare & Medicaid Services, CT computed tomography, DVT deep vein thrombosis, ECG electrocardiograph, GWTG Get With The Guidelines, HQMS Hospital Quality Monitoring System, LDL low density lipoprotein, LOS length of hospital stay, MINAP Myocardial Ischemia National Audit Project, NCDR National Cardiovascular Data Registry, NICE National Institute for Health and Care Excellence, NIHSS National Institutes of Health Stroke Scale, NSF National Service Framework, PCI percutaneous coronary intervention, SINAP Stroke Improvement National Audit Programme, SSNAP Sentinel Stroke National Audit Programme, STEMI ST-segment elevation myocardial infarction, TIA transient ischemic attack

**Table 2:** Quality improvement initiatives and achievements for acute myocardial infarction or acute ischemic stroke care, in the US, the UK and India

	The US	The UK	India
For acute myocardial infarction	**Initiatives** D2B Alliance; AHA Get With The Guidelines-CAD **Achievements** **Door-to-balloon time:** declined from a median of96 minutes in 2005, to a median of 64 minutes in2010. There were corresponding increases in the percentage of patients who had times<90 minutes (44.2% to 91.4%) and <75 minutes(27.3% to 70.4%). **Reperfusion rate**: 55% in 1990 and 94% in 2009;^16,87^ **30-day mortality**: 18.8% in 1995 and 15.8% in 2006^14^	**Initiatives** Myocardial Ischemia National Audit Project National Health Service Heart Improvement Program National Infarct Angioplasty Project **Achievements** **30-day mortality**: declined for STEMI (RR = 0.43, 95% CI: 0.34 to 0.54) and NSTEMI (RR= 0.66, 95% CI: 0.55 to 0.78) from 2003 to 2010.^88^ **Primary PCI**: 71% in 2013^89^ compared to 49% in 2009.^90^ **Primary PCI within 90 minutes of arrival:** 92.1% in 2013 compared with 52.2% in 2004.^89^	**Initiatives** STEMI India^91^ **Achievements** **Reperfusion rate**: Increase from 13% to 58% in a pilot study of TN-STEMI^92^
For acute ischemic stroke	**Initiatives** AHA Get With The Guidelines-Stroke; National Stroke Association Stroke Center Network hospital stroke program **Achievements** **Intravenous IV tPA:** 4.0% in 2003 to 7.0% in 2011.^93^ **Door-to-needle time for tPA administration:** declined from 77 minutes in 2003 to 67 minutes in 2009.^94^ **In-hospital mortality** decreased from 9.93% to 8.25% **30-day mortality:** declined 4.7% for 2011 compared with that in 1999	**Initiatives** National Sentinel Stroke Audit Sentinel Stroke National Audit Program ^95^ **Achievements** **Intravenous rt-PA:** 10.3% in 2011 to 2012,^96^ compared to 1.4% in 2008^97^	**Initiatives** The National Stroke Registry program **Achievements** None

AHA indicates American heart association, PCI percutaneous coronary intervention, STEMI ST-segment elevation myocardial infarction.

## Panel 1 Key messages

- China has recently been strengthening its health care system through the far-reaching health care reform. However, facing the huge and increasing burden of cardiovascular diseases (CVD), additional efforts are needed to ensure that individual patients are receiving care that enables them to achieve the best possible health outcomes.
- Although large progress has been made in applying new evidence, the prior studies and government reports identified substantial gaps between evidence and practice in CVD care in China, as opportunities for quality improving.
- As major barriers to quality of care, in China quality measurement system are inadequate, and there is no mechanism to link performance with the interests of care providers, despite some achievements have been made in developing a national clinical information system.
- It is urgent to expand the evidence for what works best for whom and how best to provide it in China, where medical care for CVD suffers from a deficit of local evidence about effectiveness and safety of treatments.
- Acknowledging insufficient capacities for clinical research, Chinese government has initiated long-term investments in clinical research that generate evidence for policy formulation, including the funding of Chinese National Clinical Research Centers.
- China needs to leverage existing clinical information platforms, to establish measurements based on claims data and clinical registries, for performance evaluating through measurement, improving through stronger accountable systems.
- China needs to develop national clinical research roadmap and agendas, on what is the best practice and how the performance could be improved in its own context, moreover, it is crucial to build a learning health care system engaging stakeholders, to produce actionable clinical knowledge in a more cost-effective and timely way.

## Panel 2 Literature search strategy

- To summarize the findings of large scale studies on quality of care for cardiovascular diseases in China since 2000, we did a comprehensive literature search using the databases of PubMed/Medline. We used MeSH and free text terms in conjunction to increase sensitivity to potentially appropriate studies. The MeSH terms include “Health Care Quality, Access, and Evaluation” and “Cardiovascular Diseases”. Search terms and all their possible synonyms and spellings were identified and used in the search strategy, including “China”, “treatment”, “therapy”, “healthcare” “quality”, “disparities”, “measure” “gap”, “management”, “adherence”, “equity”, “efficiency”. These terms were restricted to searching on titles and abstract fields. No restrictions were imposed on language of publications. We then further excluded letters, autobiography, comments, clinical trial, comparative study, reviews, meta-analyses and animal studies, etc.
- We also did the literature search in the database of CNKI (China National Knowledge Infrastructure) using similar strategy, to include articles published in Chinese journals. Studies were eligible for inclusion if they were large-scale observational investigations- that is, including more than 10 sites/centres with more than 1000 patients), and reported on the evaluation of in-hospital treatment in cardiovascular or cerebrovascular diseases.

## Panel 3 Engagement of stakeholders in the quality improvement and evidence generation

**Stakeholders**	**Commitments**
Government (as policy makers and health insurance agencies)	**Short-term** - Establish administrative claims data system with high-quality data andcomprehensive analysis, based on the HQMS - Establish national clinical registries focusing on quality improvement - Periodically analyse quality metrics, and make the results publicly available - Establish unique standards for electronic medical record system **Long-term** - Expand the HQMS to lower-level hospitals - Link the HQMS with other data sources - Involve standardized EMR system into regulatory requirements for hospitals
Professional societies	**Short-term** - Establish national clinical research roadmap - Issue and update clinical practice guidelines - Renew quality indicators based on updating evidence **Long-term** - Promote methodology training on clinical research
Clinical research communities	**Short-term** - Leverage the existing quality measure data - Foster sustainable collaborative research networks **Long-term** - Develop tools for dissemination and adoption of protocols and guidelines
Hospitals	**Short-term** - Establish quality workgroup and setup systems for quality monitoring - Assess the current quality status within the hospital, and set improvement goals - Assess varied quality improvement strategies for implementation **Long-term** - Develop and pilot coordination with other hospitals
Doctors	**Short-term** - Integrate the treatment strategies with context of individual patients - Empower patients for decision making - Reflect the care provided, identify the improvement opportunities **Long-term** - Apply decision support tools and patient self management tools - Ensure the integrity of medical records	
Patients	- Consent to provide information for clinical research	
